# Professional and Home-Made Face Masks Reduce Exposure to Respiratory Infections among the General Population

**DOI:** 10.1371/journal.pone.0002618

**Published:** 2008-07-09

**Authors:** Marianne van der Sande, Peter Teunis, Rob Sabel

**Affiliations:** 1 National Institute for Public Health and the Environment (RIVM), Bilthoven, Netherlands; 2 Hubert Department of Global Health, Rollins School of Public Health, Emory University, Atlanta, Georgia, United States of America; 3 Netherlands Organisation for Applied Scientific Research (TNO), Rijswijk, Netherlands; McGill University, Canada

## Abstract

**Background:**

Governments are preparing for a potential influenza pandemic. Therefore they need data to assess the possible impact of interventions. Face-masks worn by the general population could be an accessible and affordable intervention, if effective when worn under routine circumstances.

**Methodology:**

We assessed transmission reduction potential provided by personal respirators, surgical masks and home-made masks when worn during a variety of activities by healthy volunteers and a simulated patient.

**Principal Findings:**

All types of masks reduced aerosol exposure, relatively stable over time, unaffected by duration of wear or type of activity, but with a high degree of individual variation. Personal respirators were more efficient than surgical masks, which were more efficient than home-made masks. Regardless of mask type, children were less well protected. Outward protection (mask wearing by a mechanical head) was less effective than inward protection (mask wearing by healthy volunteers).

**Conclusions/Significance:**

Any type of general mask use is likely to decrease viral exposure and infection risk on a population level, in spite of imperfect fit and imperfect adherence, personal respirators providing most protection. Masks worn by patients may not offer as great a degree of protection against aerosol transmission.

## Introduction

With a potential influenza pandemic looming, governments need to decide how they can best use available resources to protect their people against severe illness and death, and to mitigate health and social effects for society as a whole. Much research is being devoted to develop optimal strategies for the use of (pre)pandemic vaccines and of anti-virals. There are only limited data to assess the potential effectiveness of non-pharmaceutical interventions to reduce the risk of transmission, including the effect of different kinds of face-masks worn by the general public or by patients.

Respiratory infections such as influenza are transmitted through infectious particles, small enough to be suspended in air [1]. Influenza transmission can occur via large droplets, which only remain suspended in the air for a short period of time thus requiring close contact, and can occur via small airborne particles, which remain suspended in air for considerable longer periods of time, and can thus be transmitted over larger distances [2]. Furthermore, some transmission may occur via direct contact with respiratory secretions such as on hands and surfaces [2].

Interruption of transmission may allow containment of major outbreaks, like pandemic influenza. Opportunistic data collected during the SARS epidemic in Asia suggested that population-wide use of face masks may significantly decrease transmission of not only SARS but also influenza [3], [4], [5], [6], [7]. As part of pandemic preparedness, many are contemplating the contribution wide-spread use of masks could have [8], [9]. As this has major implications for resource allocation and for communication, there is great need for data to guide such decisions and make them evidence-based.

Protective effects of face masks have been studied extensively, but usually this involved personal respirators for professionals under idealized conditions, because of specific applications, for instance in military or occupational uses, involving protection of specifically trained personnel. This is different from deployment of masks in the general population during an outbreak of an infectious disease, where anyone may encounter the infectious micro-organism, implying much greater heterogeneity, in training levels (experience and understanding), goodness of fit of a mask, and activities interfering with mask use and thus reducing potential reduction of transmission. The protective effect of masks is created through a combined effect of the transmission blocking potential of the material, the fit and related air leakage of the mask, and the degree of adherence to proper wearing and disposal of masks. Personal respirators such as those worn by staff attending TB patients, are used primarily to protect the wearer, and are designed to fit to the face with as tight a seal as possible. Their efficiency is graded on the degree of protection the material offers, assuming a perfect fit and optimal compliance. In contrast, surgical masks, as commonly worn in the operating theatre, are primarily used to protect the environment from the respiratory droplets produced by the wearer. With these masks, facial fit is much looser. The fit of home made masks, which could be e.g. made of a tea cloth or other comparable material available in the home, is likely to be even looser. Thus personal respirators confer a higher degree of protection than surgical masks, and these are again likely to give a higher degree of protection than home-made masks. In professional situations, ample time might be available prior to use to ensure a perfect fit and to give extensive counselling on adherence, but it is unlikely this will apply to the general population in case of a pandemic. It is possible that the discomfort in wearing associated with a certain type of masks will lead to reduced adherence and thus to a loss in overall protectiveness [10], [11]. Indeed a review among health care workers could not determine whether personal respirators conferred better protection for the health care workers than surgical masks [10].

To investigate the levels of protection, and their variation, wearing of face masks could convey to untrained subjects we designed a study in which healthy volunteers would be wearing different types of professional and home-made masks during a selection of activities, in different conditions (inward protection). We also assessed the protection different types of masks could convey when worn by a simulated infectious patient (outward protection). Resulting quantitative descriptions of distributions of protection factors may be used for assessing the importance of mask use in respiratory disease transmission.

## Methods

### Design and description of the study

Three different experiments were undertaken to assess 1) short-term protection for different types of masks worn during 10–15 minutes by the same volunteer following a standardized protocol, 2) long-term protection of a specific mask worn continuously by a volunteer for 3 hours during regular activities, and 3) effectiveness of different types of mask in preventing outgoing transmission by a simulated infectious subject. Inward protection was defined as the effect of mask wearing to protect the wearer from the environment; outward protection was defined as the effect of a mask on protecting the environment from the generation of airborne particles by a patient (or in this case a mechanical head).

**Figure 1:**
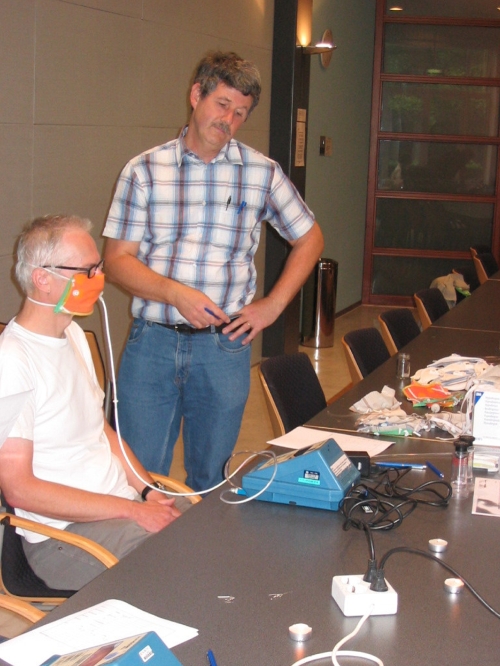
Protection factor of home-made mask being measured by Portacount in volunteer. Volunteer with home-made mask made of tea cloth. Note the candles in the foreground and the other mask types in the background.

In the first short-term experiment, 28 healthy adult volunteers were recruited, as well as 11 children between 5 and 11 years of age. Each volunteer followed the same protocol wearing a Filtering Facepiece against Particles (FFP)-2 mask 1872V® (3M); which is the European equivalent of a N95 mask, a surgical mask (1818 Tie-On®, 3M; with a filtering efficiency of around 95% for particles of sizes between 0.02 µm to 1 µm; http://jada.ada.org/cgi/content/full/136/7/877) and a home-made mask (made of TD Cerise Multi® teacloths, Blokker). In this standard protocol, the volunteer was asked to perform five successive tasks in a fixed sequence 1.5 minute of duration each: no activity-sit still, nod head (“yes”), shake head (“no”), read aloud a standard text, stationary walk. In this sequence of activities, the respiratory rate is gradually increased. Throughout this exercise, the concentration of particles was measured on both sides of the mask through a receptor fixed on the facial and on the external side. These were connected to a portable counter of all free floating particles in the air via an electrostatic particle classifier and counter, the Portacount®. The Portacount® can register particles floating in the air with sizes between 0.02 µm to 1 µm, covering most of the size range of infectious respiratory aerosols [12]. Total inward leakage (TIL) percentage was calculated by dividing the concentrations on the outside and on the inside (TIL = (concentration inside/concentration outside)×100); the calculated quantitative protection factor was the inverse of the leakage (PF = (TIL/100)^−1^). To ensure small numbers of particles produced by the volunteers would not affect measurements, we checked that at least 10,000 particles per cm^3^ particles of this size class (0.02 µm–1 µm) were present in the room which were produced by a number of lit candles. (Figure 1)

In the second long-term experiment, 22 volunteers, all adults, 10 men, 12 women, were divided into 3 groups. Each group wore a single type of mask for a period of three hours, being either a FFP2 mask (4 males, 4 females), a surgical mask (3 males, 4 females) or a home-made mask (3 males, 4 females), similar to the masks used in the short-term experiment described above. At the beginning and end of each three-hour period, full series of measurements were taken using the standardised protocol as described for the short-term experiment, and during the three hour period while wearing the masks, participants reported back at regular intervals for a short measurement during rest (absence of activity). For the remainder of the period, participants carried on with their usual daily activities. During regular activities in between measurements, the probes of the masks were plugged which did not involve dislodging of the masks.

In the final experiment, we assessed the effectiveness of different types of masks in reducing outgoing transmission from an infectious subject shedding aerosolised particles. This was simulated by fitting the different types of masks to an artificial test head, which was connected to PC-driven respirator (Bacou® LAMA AMP, Modelref 1520307). Breathing frequency was varied to mimic different respiratory rates (15, 25 and 40/minute). Only expiration was simulated; twice for each mask at each respiratory rate. The breathing flow was defined as (respiratory rate/minute x volume per breath (2 litres)) resulting in a breathing flow of 30, 50 and 80 litres per minute, which correlates with light (walking), medium (marching with backpack) and strenuous (running) activities [13]. Concentrations of particles were measured as described above by a TSI Portacount Respirator Fit tester, model 8020, measuring outward protection, rather than inward protection.

All volunteers received written information prior to the experiments and gave oral informed consent. For the children also a parent gave oral informed consent, and a parent remained present during the experiments. The Dutch Central Committee on Research Involving Human Subjects (CCMO) informed us in writing that this project did not need to be assessed by an Ethics committee.

### Data analysis

Protection factors (PF) calculated from measurements of particle concentration by Portacount® devices were reported as the ratio of particle concentrations outside and inside the mask. This is a similar concept to the fit factor as used by the US Occupational Safety and Health Administration (http://www.osha.gov/pls/oshaweb/owadisp.show_document). Therefore, a higher PF is better and PF = 1 means complete absence of protection. For statistical analysis, the following transformation was used:
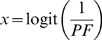
The inverse of the PF (1/PF) can be interpreted as a probability (that any particle succeeds in moving through the barrier the mask provides). The logit transformation is a standard transformation to transform the probability scale (0,1) to the real axis (-infinity, +infinity) to allow standard regression techniques (including ANOVA) to test the effects of co-variables (mask type, age class, sex, activity, duration of use) on transformed PFs in a linear model, using the statistical application R (version 2.5.0). The p-values are based on testing the ratio of mean squares for a factor (like ‘mask’) and the mean square of errors (random fluctuations), assuming that ratio is F-distributed. Whenever the p-value (the probability of a greater value of the tested ratio) is greater than 0.05, the ratio is considered significantly different from 1 ( = indifference) at the 95% level.

## Results

### Short term inward protection experiment

All masks provided protection against transmission by reducing exposure during all types of activities, for both children and adults (Table 1). Within each category of masks, the degree of protection varied by age category and to a lesser extent by activity. We observed no difference between men and women. Surgical masks provided about twice as much protection as home made masks, the difference a bit more marked among adults. FFP2 masks provided adults with about 50 times as much protection as home made masks, and 25 times as much protection as surgical masks. The increase in protection for children was less marked, about 10 times as much protection by FFP2 versus home-made masks and 6 times as much protection as surgical masks.

**Table 1:** Median (IQR) protection factor by mask, by activity, by age category.

		no activity	nodding	shaking	reading	walking
Tea cloth	Adults	2.5 (2.1–2.9)	2.2 (1.9–2.5)	2.2 (1.9–2.7)	3.2 (2.5–3.9)	2.4 (2.1–3.3)
	children	2.2 (1.5–2.2)	1.9 (1.5–2.3)	1.9 (1.4–2.3)	2.2 (1.8–3.7)	2.2 (1.8–2.4)
Surgical mask	Adults	4.1 (3.1–7.2)	4.7 (3.4–7.3)	5.1 (3.2–7.6)	5.3 (4.3–8.0)	4.2 (3.1–5.7)
	children	3.2 (2.2–4.1)	3.4 (2.7–5.2)	3.6 (2.7–4.3)	4.9 (4.0–5.3)	3.6 (2.4–4.2)
FFP2 mask	Adults	113 (26–210)	82 (45–179)	91 (23–187)	66 (29–107)	99 (19–169)
	children	18 (6.1–165)	13 (3.8–41)	18 (4.0–54)	35 (8.6–91)	15 (5.1–176)

IQR = interquartile range

In these short term experiments, adjusting for covariates, face mask type had a strongly significant independent effect on protection (p<0.001). Children were significantly less protected than adults (p<0.001). There was no significant impact of activity on protection.

### Long term inward protection experiment

**Figure 2:**
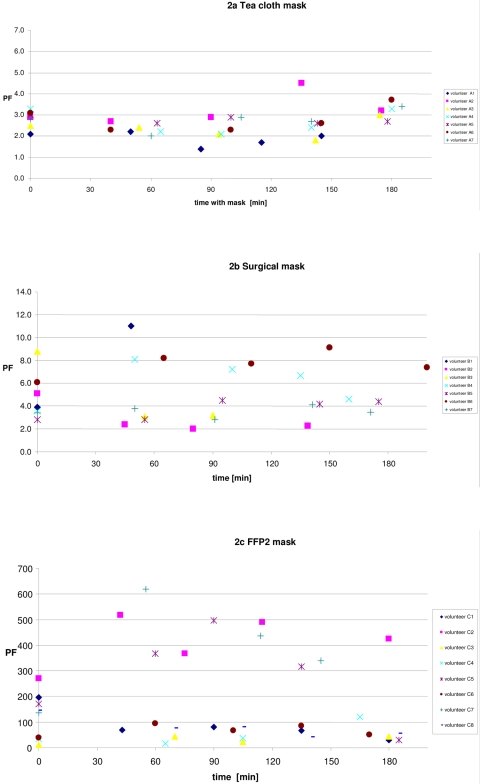
Protection factors over time per volunteer by type of mask worn. Please note different scale on Y-axis!

As in the short term experiment, mask type was a strong determinant of protection (Table 2). Protection factors for each type of mask were similar to the protection factors measured in the short term experiments for adults. There was considerable variability between volunteers. The median protection factors measured over a 3 hour period increased for those wearing home-made masks, decreased for those wearing FFP2 masks, and did not show a consistent pattern for those wearing a surgical mask (Figure 2), but overall protection factors calculated per type of mask were stable over time, and did not change statistically significant with prolonged wearing. Overall, protection factors were relatively stable over time for each individual (ANOVA p = 0.4). Males and females did not have significantly different protection factors (ANOVA p = 0.9). As in the short term experiment, protection conferred by surgical masks was higher than protection given by a home-made mask, and protection provided by a FFP2 masks was again markedly higher than protection provided by a surgical mask. As in the short term experiment, more strenuous activities (reading and walking) tended to increase the protection of the home-made mask and to a lesser extent of the surgical mask, and decreased the protection by the FFP2 mask, but there was no overall significant effect of type of activity on PF (ANOVA p = 0.1).

**Table 2:** Median (IQR) protection factors at start and end of long term-experiment, by mask, by activity.

		no activity	nodding	shaking	reading	walking
Tea cloth	Start	2.8 (2.5–3.1)	2.4 (2.3–2.6)	2.5 (2.3–2.8)	3.4 (2.9–3.7)	2.4 (2.2–3.1)
	End	3.2 (2.7–3.4)	2.7 (2.5–3.0)	2.9 (2.6–3.4)	4.3 (3.5–5.2)	2.9 (2.8–2.9)
Surgical mask	Start	3.9 (3.4–6.1)	3.6 (3.1–7.1)	3.8 (3.7–7.3)	6.5 (4.3–7.2)	4.6 (2.9–6.4)
	End	4.4 (3.2–7.4)	4.5 (3.4–7.2)	4.1 (3.3–7.8)	5.9 (4.2–6.5)	3.9 (3.3–6.7)
FFP2 mask	Start	141 (34–196)	100 (26–156)	132 (54–265)	84 (47–194)	79 (10–167)
	End	53 (31–339)	48 (36–116)	42 (23–177)	92 (29–202)	43 (16–185)

IQR = interquartile range

### Outward protection experiment

**Figure 3:**
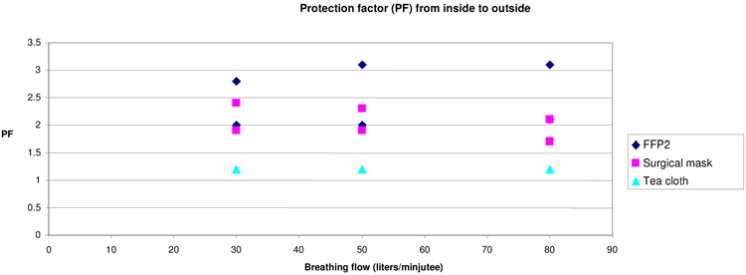
Outward protection factors at a range of breathing flows for a mechanical head with different types of masks, with two meaurements per mask at each breathing flow. PFs for teacloth did not differ during the repeated measurement at each breathing flow, so light blue triangles overlap in figure.

In a final experiment, retention of particles expelled inside the masks was studied. Here again, mask type was strongly correlated with (transformed) protection factors. Protection factors for all type of masks were considerably lower than those observed for inward protection. The home-made masks only provided marginal protection, while protection offered by a surgical mask and an FFP2 mask did not differ (figure 3).

The simulated breathing frequency did not significantly affect the measured protection factors. Adjusting for covariates, mask type and particle concentration, but not flow rate, were significant factors for protection in the reverse flow experiment.

## Discussion

In our experiments, the main determinant of the magnitude of protection factors measured by masks was the type of mask, which can be seen as a proxy for potential reduction in infectious disease transmission. The duration of wear and the type of activity did not have a significant impact on exposure reduction. Thus, the expected superior protection conferred by a professional FFP2 mask compared to a surgical mask or a home-made mask was maintained when these FFP2 masks were worn by healthy lay people in spite of the increased risk of a poor fit and significant behavioural leakage.

Children were significantly less protected from exposure than adults, which might be related to an inferior fit of the masks on their smaller faces. Although we observed a high degree of individual variability in the degree of protection conferred as reflected in the wide interquartile ranges of the measured PFs, no systematic difference was found between men and women, suggesting a poorer fit only has a noticeable impact on protection when the mismatch between face and mask is considerable. All types of masks provided a much higher degree of exposure protection against inward transmission of particles, then in preventing outward transmission by a mechanical head as a proxy for an infected patient exposing the environment.

Data from professional users suggest a decrease in protection over time due to a reduction in fibre charges [13]. In our data, this effect was not significantly present, although a tendency towards reduced protection over time was seen for the FFP2 masks. Also, our study showed a high degree of individual variation in exposure protection. This is important as it reflects the presence of many different sources of variation, behavioural as well as anatomical, which can also be expected to be present if the general population would be requested to wear face masks in case of a pandemic. Furthermore, we do not know from these experiments whether reduced exposure has a linear or non-linear relationship to the reduction of infection risk.

Although this could imply that individual subjects may not always be optimally protected, from a public health point of view, any type of general face mask usage can still decrease viral transmission. Also, it is important not to focus on a single intervention in case of a pandemic, but to integrate all effective interventions for optimal protection.

Surprisingly, the protection conferred by each of the masks appeared stable over time and was not dependent on activity. This suggests that leakage associated with suboptimal fit and compliance was stable over time. The tendency towards improved protection of the poorer fitting masks with increased activities such as reading, might be attributable to reduced leakage when breathing through the mouth rather than the nose, which could give some overpressure and thus reduce inward leakage. We had assumed that compliance would decrease during the three hours of continuous wearing, in particular with more strenuous activities. Indeed, among professionals like cullers, there have been some anecdotal reports that FFP3 masks were associated with poorer compliance than FFP2 masks in wearing. Where a reduction in protection was found with the FFP2 mask, the reverse was seen for the home-made mask. It is possible that the experimental situation, sufficient motivation to endure a relatively limited time of discomfort, and the absence of physically challenging activities, has provided more stable protection than might be found in real-life situations. However, overall these experiments show that significant protection against influenza transmission upon exposure can be conveyed also for lay people, including children, in spite of imperfect fit and imperfect adherence.

It is also clear that home-made masks such as teacloths may still confer a significant degree of protection, albeit less strong than surgical masks or FFP2 masks. Home made masks however would not suffer from limited supplies, and would not need additional resources to provide at large scale. Home made masks, and to a lesser degree surgical masks, are unlikely to confer much protection against transmission of small particles like droplet nuclei, but as the reproduction number of influenza may not be very high [14] a small reduction in transmissibility of the virus may be sufficient for reducing the reproduction number to a value smaller than 1 and thus extinguishing the epidemic [15]. Greater reduction in transmissibility may be achieved if transmission is predominantly carried by larger droplets. In a typical human cough half of the droplets may be small (<10 µm), but these comprise only a small fraction (2.5*10^−6^) of the expelled volume [12]. Smaller droplets may however more easily penetrate the smaller bronchi and be more effective in transmission [1]. A more detailed analysis of aerosol and droplet inoculation and infectivity may provide better insight into the impact of either transmission mode on population spread.

The difference in measured protection against inward and outward protection is remarkable, and cannot be explained from the available data as we only measured the overall effect. A differential effect on the amount of leakage seems most plausible. At the same time, we cannot exclude that wearing of face masks, even FFP2 or surgical masks by patients might still significantly reduce transmission. However, the observed limited particle retention in our experiments may still be an overestimate of protection, as it may for instance be challenging to enforce adherence to mask wearing by a patient who is short of breath. Wearing of masks by caregivers might be more feasible and more effective, in particular where additional preventive measures are in place as well for caregivers.

Furthermore, we should bear in mind that this is an experimental study, with relatively small numbers of volunteers, which limits the generalisability of some of our findings. E.g., for masks to have any impact during an actual pandemic, people may need to be wearing masks during several weeks with many shorter or longer mask-free periods. Furthermore, the PFs may be an over- or underestimation of the actual protection conferred. And although our simulated patient varied its breathing frequency, we have not assessed the impact of e.g. coughing or sneezing on outward transmission through a mask.

A recent analysis of the 1918 epidemic, noted that cities where strict interventions were implemented early on to prevent transmission, were overall worse-off than cities where some degree of transmission occurred early on [16]. Given the need for the population to acquire sufficient natural immunity over time, it can not be excluded that the amount of protection conferred by home made masks might sufficiently reduce viral exposure to impact on transmission during the early waves, while allowing people enough exposure to start mounting an efficient immune response. Further field studies are needed to assess acceptability and effectiveness of masks worn by people from the general population. Also, experimental data are needed to develop dose-response models which may improve understanding of determinants of transmission. A cost-effectiveness analysis might give further insights in the relative benefits of home made masks.
